# Effects of Low-Calorie Sweetener Restriction on Glycemic Variability and Cardiometabolic Health in Children with Type 1 Diabetes: Findings of a Pilot and Feasibility Study

**DOI:** 10.3390/nu15183867

**Published:** 2023-09-05

**Authors:** Allison C. Sylvetsky, Hailey R. Moore, Xinyu Zhu, Jasmine H. Kaidbey, Leyi Kang, Abbas Saeed, Shazmeena Khattak, Mariana F. Grilo, Natalie Vallone, Janae Kuttamperoor, Fran R. Cogen, Angelo Elmi, Peter J. Walter, Hongyi Cai, Loretta DiPietro, Michael I. Goran, Randi Streisand

**Affiliations:** 1 Department of Exercise and Nutrition Sciences, Milken Institute School of Public Health, The George Washington University, 950 New Hampshire Avenue NW, Suite 200, Washington, DC 20052, USA; kaidbey@gwmail.gwu.edu (J.H.K.); aasaeed21@gmail.com (A.S.); shazmeena.khattak@gmail.com (S.K.); marianafg@gwmail.gwu.edu (M.F.G.); natalie.vallone@email.gwu.edu (N.V.); janaekuttamp@email.gwu.edu (J.K.); ldp1@gwu.edu (L.D.); 2 Division of Psychology & Behavioral Health, Children’s National Hospital, 111 Michigan Avenue NW, Washington, DC 20010, USA; hmoore2@childrensnational.org (H.R.M.); lkang1@childrensnational.org (L.K.); rstreis@childrensnational.org (R.S.); 3 Nutrition and Health Sciences Program, Emory University, 1518 Clifton Rd, Atlanta, GA 30322, USA; xinyu.zhu@emory.edu; 4 Division of Endocrinology, Children’s National Hospital, 111 Michigan Avenue NW, Washington, DC 20010, USA; fcogen@childrensnational.org; 5 School of Medicine and Health Sciences, The George Washington University, 2300 I St. NW, Washington, DC 20052, USA; 6 Department of Biostatistics and Bioinformatics, Milken Institute School of Public Health, The George Washington University, 950 New Hampshire Avenue NW, Suite 200, Washington, DC 20052, USA; afelmi@gwu.edu; 7 Clinical Mass Spectrometry Lab, National Institute of Diabetes, Digestive and Kidney Diseases (NIDDK), National Institutes of Health (NIH), 9000 Rockville Pike, Bethesda, MD 20892, USA; walterpj@niddk.nih.gov (P.J.W.); hongyi.cai@nih.gov (H.C.); 8 Department of Pediatrics, The Saban Research Institute, Children’s Hospital Los Angeles, 4650 Sunset Blvd, Los Angeles, CA 90027, USA; goran@usc.edu

**Keywords:** beverages, artificial sweetener, sugar substitutes, glycemic control, metabolism, diet soda, sugar

## Abstract

Low-calorie sweeteners (LCS) are commonly consumed by children with type 1 diabetes (T1D), yet their role in cardiometabolic health is unclear. This study examined the feasibility, acceptability, and preliminary effects of 12 weeks of LCS restriction among children with T1D. Children (*n* = 31) with T1D completed a two-week run-in (*n* = 28) and were randomly assigned to avoid LCS (LCS restriction, *n* = 15) or continue their usual LCS intake (*n* = 13). Feasibility was assessed using recruitment, retention, and adherence rates percentages. Acceptability was assessed through parents completing a qualitative interview (subset, *n* = 15) and a satisfaction survey at follow-up. Preliminary outcomes were between-group differences in change in average daily time-in-range (TIR) over 12 weeks (primary), and other measures of glycemic variability, lipids, inflammatory biomarkers, visceral adiposity, and dietary intake (secondary). Linear regression, unadjusted and adjusted for age, sex, race, and change in BMI, was used to compare mean changes in all outcomes between groups. LCS restriction was feasible and acceptable. No between-group differences in change in TIR or other measures of glycemic variability were observed. However, significant decreases in TNF-alpha (−0.23 ± 0.08 pg/mL) and improvements in cholesterol (−0.31 ± 0.18 mmol/L) and LDL (−0.60 ± 0.39 mmol/L) were observed with usual LCS intake, compared with LCS restriction. Those randomized to LCS restriction did not report increases in total or added sugar intake, and lower energy intake was reported in both groups (−190.8 ± 106.40 kcal LCS restriction, −245.3 ± 112.90 kcal usual LCS intake group). Decreases in percent energy from carbohydrates (−8.5 ± 2.61) and increases in percent energy from protein (3.2 ± 1.16) and fat (5.2 ± 2.02) were reported with usual LCS intake compared with LCS restriction. Twelve weeks of LCS restriction did not compromise glycemic variability or cardiometabolic outcomes in this small sample of youth with T1D. Further examination of LCS restriction among children with T1D is warranted.

## 1. Introduction

Type 1 diabetes (T1D) affects approximately 1 in 500 children and requires careful management to prevent or delay the development of diabetes complications [1]. A common nutritional strategy for diabetes management is the replacement of added sugars with low-calorie sweeteners (LCS) [2] such as aspartame, sucralose, saccharin, stevia, and acesulfame-potassium (ace-K). While LCS are a heterogeneous group of compounds with different physical and chemical properties, they all provide sweetness with no or few calories at a reduced glycemic load. While the use of LCS in place of added sugars reduces the calorie and sugar content of foods and beverages, the role of LCS in cardiometabolic health and their impacts on the overall diet are not well understood [3].

Although LCS may be useful for reducing energy intake and managing weight in the short term [4], long-term consumption of LCS is associated with an increased risk of chronic diseases [5], such as cardiovascular disease [6], stroke [7], and cancer [8], as well as higher all-cause mortality [9], in prospective cohort studies in adults without diabetes. While data from long-term randomized controlled trials (RCTs) in humans are lacking [9], findings of mechanistic studies [10] and small, relatively short-term RCTs [3] have also demonstrated that chronic LCS consumption may unfavorably impact cardiometabolic risk factors. LCS are metabolically active and are proposed to act through a variety of potential mechanisms [11] including the disruption of glucose-insulin homeostasis [12,13,14,15], disturbance of gut microbiota [16,17,18], and dysregulation of inflammatory pathways [19,20,21]. However, despite their widespread use among children [22,23], LCS consumption in children with T1D is severely understudied. While several studies have examined LCS consumption among children without diabetes in relation to differences in weight and/or adiposity [24], there is a dearth of evidence investigating the effects of LCS consumption on other metabolic outcomes among youth [25].

Herein, we report the feasibility, acceptability, and preliminary effects of DRINK-T1D [26], a pilot RCT designed to investigate the effects of 12 weeks of LCS restriction (replacement of usual LCS-containing beverage consumption with unsweetened water and avoidance of other sources of LCS) on glycemic variability, lipid profiles, systemic inflammation, and visceral adiposity, compared with continuation of usual LCS intake. We hypothesized that the average daily time in the target glycemic range (TIR) would be increased and HbA1c reduced after 12 weeks of LCS restriction, compared with after 12 weeks of usual LCS consumption. We further hypothesized that lipid profiles would be improved, and circulating concentrations of inflammatory biomarkers reduced, in the intervention group (LCS restriction), compared with the control group (usual LCS intake).

## 2. Materials and Methods

The DRINK-T1D study was registered on ClinicalTrials.gov (NCT0438588, Effects of Low-calorie Sweetened Beverage Restriction in Youth with Type 1 Diabetes) and the study was conducted according to the guidelines of the Declaration of Helsinki and approved by the Institutional Review Board at Children’s National Hospital (protocol 000012436, approved 21 February 2020) prior to beginning the study procedures.

### 2.1. Study Participants

Children with T1D were recruited from the *Washington Nationals* Diabetes Care Complex (DCC) at Children’s National Hospital (CNH) in Washington D.C., as well as the pediatric diabetes clinic at Pediatric Specialists of Virginia (PSV) in Fairfax, VA, and through sending announcements on local listservs for children with T1D and their parents. For children recruited from CNH, a trained research assistant (RA) reviewed clinic schedules weekly and sent a recruitment letter to families with potentially eligible children, who were identified using medical records. For children recruited at PSV or via local listservs, parents contacted the study team using the information provided in the study advertisement. Recruitment took place from August 2020 to December 2022. While most recruitment took place virtually, due to the COVID-19 pandemic, children seen at CNH were also recruited in person in the clinic waiting room. Inclusion criteria were age between 5 and 14 years old, having physician-diagnosed T1D for at least 12 months, already using a DEXCOM G6 continuous glucose monitor (CGM), and reporting habitual intake of at least 12 ounces of LCS-containing beverages per day. Exclusion criteria were the use of medications impacting cardiometabolic outcomes, other than insulin. Eligibility was determined via telephone, using a brief study eligibility checklist, which included several questions about the child’s usual LCS consumption. For example, parents were asked, “does your child consume diet, sugar-free, or reduced-sugar beverages such as diet soda, light fruit juice, diet or “zero” sports drinks, or diet iced tea”? If the parent responded “yes”, they were asked to provide additional information about the brand, frequency, and quantity of consumption for each LCS-containing beverage their child consumed. Analogous questions were subsequently asked to assess the child’s consumption of LCS-containing foods, condiments, and packets.

### 2.2. Study Procedures

#### 2.2.1. Virtual Enrollment Visit

Upon enrollment, participants attended a virtual enrollment visit, where informed consent and assent were electronically obtained from the parent and child, respectively. Sociodemographic information was obtained via a parent report, after which, the parent was instructed to complete several questionnaires, including the Diabetes Self Care Inventory (SCI) [27], Patient Reported Outcomes Measurement Information System—Physical Activity Survey (PROMIS-PA) [28], and a LCS intake questionnaire, which has not been validated, but was developed by the study team to assess the child’s habitual intake of beverages, foods, condiments, and packets containing LCS. For each LCS-containing item reported, participants were asked to estimate the frequency (never, once per month or less, 2–3 times per month, once a week, 2–3 per week, and once per day or more) of consumption and the amount they typically consumed. The frequencies and amounts reported were converted into a number of servings per day. At the end of the virtual enrollment visit, participants were instructed to continue their usual LCS intake and CGM use and complete a 7-day photo-assisted food record during the week prior to their baseline study visit, which was scheduled for at least two weeks later. The two-week period between the virtual enrollment visit and baseline visit allowed for 14 days of CGM data collection prior to randomization.

#### 2.2.2. Baseline Study Visit

The baseline study visit took place in person at CNH. The child’s height, weight, and vital signs were measured using standard procedures, and a spot urine sample was collected for measurement of urinary LCS concentrations. A fasted blood sample was then collected by a research nurse and a subset of participants (*n* = 8, due to COVID-19-related staffing issues, which restricted access to radiology resources) also underwent dual X-ray absorptiometry (DXA) for the assessment of visceral adiposity. The child’s CGM data for the prior two weeks were downloaded using Dexcom Clarity v3.46.1 software.

#### 2.2.3. Randomization to LCS Restriction or Usual LCS Intake

Children were then randomized to either LCS restriction or continuation of usual LCS intake (control) for 12 weeks. Children randomized to LCS restriction were provided with a short brochure on avoiding LCS, which included a list of LCS-containing foods and beverages to avoid throughout the study, and were also provided with samples of still and seltzer water to encourage adherence. Children randomized to the control group were instructed to continue their usual LCS intake, consistent with standard clinical guidance provided by dietitians in the DCC, and were given a variety of sample LCS-containing beverages to take home. Participants in both groups also received brief information on general healthy eating strategies, consistent with standard dietary guidance provided by dietitians at CNH. Treatment-group-specific study instructions were reinforced by text messages sent throughout the study and by the RA mid-way through the intervention period (approximately 6 weeks after randomization) during a virtual booster visit, which took place via Zoom™ Zoom Video Communications, Inc., San Jose, CA, USA.

#### 2.2.4. Adherence to Treatment Assignment

Adherence to the treatment assignment was monitored using parent-reported responses to LCS consumption surveys sent daily via RedCap™ Vanderbilt University, Nashville, USA throughout the study, and objectively, through the collection of spot urine samples for the measurement of LCS (specifically sucralose and ace-K) concentrations at baseline and week 12. While some LCS-containing beverages consumed by participants were sweetened with aspartame, it was not possible to measure aspartame concentrations in the urine due to its rapid degradation following ingestion [29].

#### 2.2.5. 12-Week Follow-Up Visit

The 12-week follow-up visit took place at CNH and was scheduled at approximately the same time of day as the participant’s baseline visit. During the week prior to the visit, participants were again instructed to complete a photo-assisted, 7-day food record. During the 12-week follow-up visit, a blood sample was collected for the assessment of metabolic and inflammatory biomarkers and the child’s CGM data from the past 14 days were downloaded. A DXA scan was also performed if the child had a DXA scan at baseline. At the end of the visit, the participants’ parent was asked to complete a brief satisfaction survey and a subset of parents (*n* = 15, parents of consecutive participants until saturation of the data was reached) was invited to participate in a qualitative interview about their experience in the study and any challenges they encountered. Qualitative interviews were conducted by a trained study team member using a semi-structured interview guide and lasted 15–20 minutes.

#### 2.2.6. Laboratory Assays and Dietary Data Collection

Additional details of the study procedures, including laboratory assays, questionnaires, and food record completion have been previously published in a manuscript describing the rationale and design of the study [26]. Liquid chromatography-mass spectrometry (LC-MS) instruments were upgraded to a Thermo Scientific Vanquish UPLC coupled with an Orbitrap ID-X mass spectrometer (Thermo Scientific, Waltham, MA, USA), performed in negative ion mode. A Waters Acquity UPLC BEH C18 column (2.1 mm × 100 mm, 1.7 µm) was used and maintained at 35 °C. Mobile phase containing solvent A (H_2_O with 0.1% NH_3_) and B (ACN with 0.1% NH_3_) at a flow rate of 300 µL/min, *d*_6_-sucralose and *d*_4_-ace-K were used as internal standards. Calibration exceeded FDA LC-MS guidelines for linearity and quantitation.

### 2.3. Statistical Analyses

Descriptive statistics were used to summarize the participants’ demographic characteristics a comma responses to survey questions and assess the distribution of all outcomes. Qualitative interviews were transcribed and coded using thematic analysis and emergent themes and subthemes were identified, after which representative quotations were selected. Linear regression of the pre-post within-subject difference, unadjusted and subsequently adjusted for age, sex, race, and change in BMI, was used to compare the mean changes in metabolic, anthropometric, and dietary outcomes within and between groups. Participants with <7 days of CGM data at either timepoint (*n* = 3) were excluded from analyses of CGM outcomes. Main analyses were performed using an intention-to-treat (ITT) approach and a sensitivity analysis was subsequently conducted excluding non-adherent (*n* = 2) participants. All analyses were conducted using Microsoft Excel or R, using a two-sided α-level of 0.05. Because no prior study has assessed glycemic variability before and after LCS restriction, the study was powered using changes before and after LCS consumption in seven volunteers consuming saccharin (equivalent to 4–5 diet sodas per day) for 1 week [17]. Based on this effect size, 13 participants per group were required to provide 80% power to detect differences in glycemic control between the treatment groups. Further details of the sample size calculations were previously described [26].

## 3. Results

### 3.1. Feasibility

**Figure 1:**
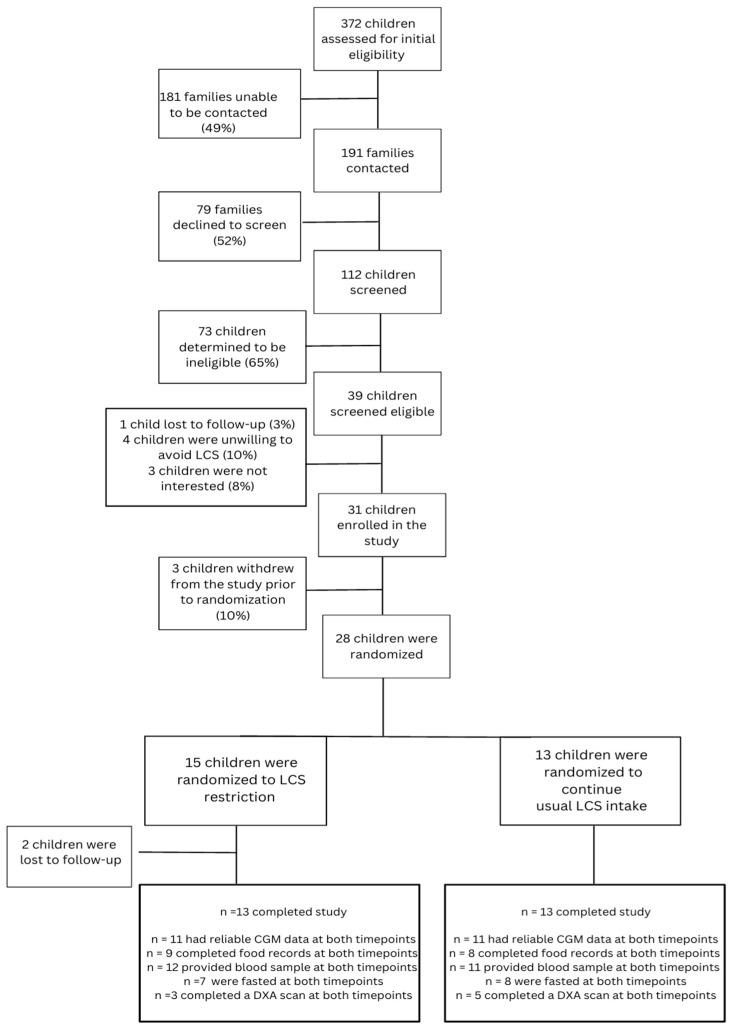
Participant recruitment, screening, enrollment, and data completion.

As shown in Figure 1, recruitment letters describing the study were mailed to 372 families with a potentially eligible child, who were then contacted by phone approximately one week later by a RA. One hundred and eighty-one families were unable to be reached and seventy-three were determined to be ineligible (e.g., did not consume LCS, diagnosed with T1D within the past year). Of 39 families with children determined to be eligible, 32 agreed to participate (82%) and 31 enrolled in the study. Of the 31 children who enrolled in the study, 28 completed the two-week run-in period and were randomized to LCS restriction (*n* = 15) or usual LCS intake (*n* = 13). Twenty-six children (84% of those enrolled) completed the 12-week study protocol (*n* = 13 per group).

The baseline characteristics of the 28 children randomized to LCS restriction or usual LCS intake are shown in Table 1. The mean age of the children was 9.7 years, and the sample was comprised of half males and half females. Approximately half of the participants were Black or mixed race, and approximately half were White; most participants (93%) were non-Hispanic. Per inclusion criteria, all participants reported habitual intake of LCS prior to enrollment, with similar consumption between the groups. There were no statistically significant differences in baseline characteristics of participants randomized to LCS restriction or usual LCS intake.

### 3.2. Acceptability

The acceptability of the study was high, irrespective of whether participants were randomized to LCS restriction or usual LCS intake. In the satisfaction survey, most parents reported they would suggest the study to other parents of children with T1D and indicated agreement (“agree” or “strongly agree”) about the study having a positive impact on their child’s diabetes management, the study having reasonable expectations, and feeling glad that they participated (Table 2). Most parents also reported that they liked (somewhat liked or liked very much) the length of the study and the number and length of the study visits, as well as the frequency of text messages and the scheduling process. All parents indicated they liked the format and delivery of in-person, phone, and online study visits.

Parents who completed an in-depth qualitative interview also provided overall positive feedback about their study experience, indicative of high acceptability (Table 3). Two overarching themes were identified: facilitators of study participation and challenges of study participation. Although adherence to the study protocol was not perceived as being difficult, a key challenge reported by parents in both groups was the completion of food records throughout the study. Some parents of children randomized to LCS restriction also explained that it was difficult to identify which products their child needed to avoid and/or that their child did not like being limited to drinking unsweetened beverages. Benefits associated with study participation were also identified as an additional minor theme, as some parents described other positive aspects of study participation, such as that the completion of food logs encouraged more thoughtful eating habits. Some parents also mentioned that study participation gave them better control over their child’s blood sugar levels and/or encouraged their child to have greater independence regarding their diabetes management.

### 3.3. Adherence

Adherence to the completion of daily LCS consumption surveys was high in both groups, as participants completed an average of 94% and 80% of surveys sent to them (email or text, per participant preference) in the LCS restriction and usual LCS intake groups, respectively. Adherence to instructions to consume or avoid LCS per randomization was also high, with those randomized to continue usual LCS intake reporting LCS consumption on an average of 84% of the daily surveys completed throughout the study. Among those randomized to LCS restriction, all but one participant (92%) indicated not consuming any LCS on at least 85% of the daily surveys. Mean urinary concentrations of sucralose and ace-K were also markedly lower in the LCS restriction group after (14 ± 7.4 ng/mL sucralose; 698 ± 453 ng/mL ace-K, after excluding non-adherent participant) versus before (1330 ± 705 ng/mL sucralose; 40,118 ± 14,919 ace-K) the intervention and increased between baseline (654 ± 289 ng/mL sucralose; 28,925 ± 12,630 ng/mL ace-K) and 12 weeks (1242 ± 664 ng/mL sucralose; 68,335 ± 37,593 ng/mL ace-K, after excluding non-adherent participant) among those randomized to continue the usual LCS intake, increasing confidence that participants were overall adherent to their randomization assignment (Supplementary Table S1).

### 3.4. Glycemic Variability and Cardiometabolic Outcomes

No differences in unadjusted or adjusted changes in measures of glycemic variability, including time-in-range, time-above-range, time-below-range, average glucose, standard deviation of glucose, or HbA1c were observed by time or treatment group (Table 4). However, total cholesterol and TNF-alpha significantly decreased in the usual LCS intake group compared to those randomized to LCS restriction, before and after adjustment for relevant covariates. LDL also decreased in the usual LCS intake group compared with those randomized to LCS restriction in fully adjusted models and within-subjects decreases in triglycerides and TNF-alpha were observed in the usual LCS intake group. In sensitivity analyses removing two non-adherent participants (Supplementary Table S2), results were similar to the main analyses, except the difference in the change in LDL was no longer statistically significant between the groups. Among the small subset of participants who had a DXA scan at both timepoints, no differences in changes in percentage body fat or visceral fat mass were observed between children randomized to LCS restriction or usual LCS intake (Supplementary Table S3).

### 3.5. Dietary Intake

A decrease in total daily energy intake was reported in both the LCS restriction and usual LCS intake groups and was not statistically significant between the groups (Table 5). Decreases in the percentage of total calories from carbohydrates and increases in the percentage of total calories from fat and protein were also reported in the usual LCS intake group, but the between-group difference in percentage of total calories from protein was attenuated after adjustment for relevant covariates. Notably, those randomized to LCS restriction did not report increases in total sugar or added sugar intake during the study. Results of sensitivity analyses removing non-adherent participants (Supplementary Table S4) were similar to the main analyses, except the between-group difference in the change in the percentage of calories from dietary fat was attenuated in fully adjusted models.

## 4. Discussion

A pilot intervention involving 12 weeks of LCS restriction or continuation of usual LCS intake among children with T1D was highly acceptable, albeit only somewhat feasible. Participant recruitment was particularly challenging, with twelve patients approached for every one participant ultimately enrolled in the study. Response, recruitment, and retention rates were all lower than anticipated, considering that rates of trial participation among children with T1D seen at Children’s National are typically higher than in the present study [30,31].

These challenges were likely in large part attributable to conducting the entire study during the COVID-19 pandemic, while lockdown restrictions were in place. Stay-at-home orders and other restrictions related to the COVID-19 pandemic necessitated reliance on primarily remote, as opposed to in-clinic, recruitment approaches (letters and phone calls) and led to some eligible participants declining to participate or indicating hesitance to complete in-person study procedures. This posed a barrier to our ability to recruit and retain the planned sample size and contributed to higher-than-expected rates of incomplete or missing data for several of the study outcomes. Further, the relatively burdensome nature of some of the procedures (e.g., two in-person study visits with blood draws, completion of daily LCS intake surveys and repeated 7-day, photo-assisted food records) may have also detracted from study engagement.

However, adherence to treatment allocation and satisfaction with study participation was high in both groups. Participants reported having an overall positive experience in the study, with parents of children randomized to both the LCS restriction and usual LCS intake groups indicating that participation contributed to improvements in their child’s overall diabetes management and made them more aware of their child’s diet. Such changes in diabetes management behaviors during the study, however, may have contributed to the lack of between-group differences observed in most of the study outcomes. For example, increased parental awareness of their child’s diet may have contributed to the reductions in total daily energy intake reported during week 12 compared to baseline among participants in both groups period. Improvements in dietary intake during the study may also have contributed to the lack of differences in glycemic and metabolic outcomes.

The present pilot findings demonstrate that 12 weeks of LCS restriction did not improve glycemic variability or other metabolic or inflammatory biomarkers in children with T1D. These null findings are in contrast with our initial hypothesis that 12 weeks of LCS restriction would improve glycemic variability and other cardiometabolic biomarkers in children with T1D, which was based on results of several, mostly small trials in adults demonstrating that LCS consumption promotes glucose intolerance [32], reduces insulin sensitivity [14,15,33], disturbs the gut microbiota [17,32], and dysregulates inflammatory pathways [19]. Notably, the unfavorable metabolic effects of LCS consumption reported in the aforementioned studies were observed following repeated ingestion of sucralose, whereas children with T1D in the present study consumed a variety of LCS-containing products, which contain a variety of different LCS. Given that LCS are a heterogeneous group of compounds (e.g., sucralose, aspartame, acesulfame-potassium) [29], they may have divergent effects on metabolic health, which may also have contributed to the null findings.

The lack of a difference in glycemic outcomes following LCS restriction compared with usual LCS intake is consistent with findings of a 12-week intervention study in healthy adults, among whom effects of aspartame-sweetened beverage consumption were similar to water [34]. Our findings are also consistent with a recent 4-week intervention study comparing the effects of four different LCS (saccharin, sucralose, aspartame + acesulfame-potassium) with unsweetened water among healthy adult women, in which no significant differences in glycemic outcomes were reported [35]. While reductions in total cholesterol and LDL and improvements in TNF-alpha were observed in those randomized to usual LCS intake in the present study, participants in the control group were more metabolically at-risk at baseline (e.g., higher concentrations of inflammatory biomarkers, poorer glycemic control although not statistically significant) and these findings should be cautiously interpreted due to the small sample size.

While the percentage of daily energy from carbohydrates decreased in the usual LCS intake group relative to the LCS restriction group, it is notable that children randomized to LCS restriction did not report increases in energy, total sugar, or added sugar intake at follow-up compared to baseline. This demonstrates that removing LCS from the diet of children with T1D does not result in the replacement of LCS with added sugars and challenges the necessity of current clinical guidance for children with T1D to replace added sugars with LCS. This is especially important given continued uncertainty about the health effects of LCS consumption among children and the recent identification of aspartame as a “possible carcinogen” by the World Health Organization’s (WHO) International Agency for Research on Cancer (IARC) [36]. Furthermore, the fact that glycemic variability was similar following 12 weeks of LCS restriction compared with the continuation of usual LCS intake challenges whether reliance on LCS as a strategy to optimize blood glucose levels among children with T1D is justified. This warrants further consideration given a lack of available research on the effects of LCS among children [25] and concerns about their long-term health effects [9], particularly among youth [37].

A key limitation of our study was the small sample size, which limits its generalizability and may have precluded the ability to detect differences in glycemic and metabolic outcomes. The lack of complete CGM, metabolic, and/or dietary data available for several participants also further limited our ability to make robust statistical comparisons. Furthermore, due to the small sample size, variety of different LCS-containing products habitually consumed by participants in the study, use of a non-validated instrument to estimate usual LCS exposure, and lack of publicly available information available on the quantities of LCS in products, we were unable to separately examine the effects of LCS restriction by type of LCS (e.g., sucralose vs. aspartame) or by the amount of LCS (total or by sweetener) consumed, which may have contributed to the lack of an effect of LCS restriction on the study outcomes. In addition, while the measurement of urinary sucralose and ace-K concentrations as an objective marker of adherence was a strength of the study, aspartame was unable to be measured due to its rapid degradation following ingestion [29]. Urine measurements were also taken from spot urine samples (as opposed to 24-h urine), which provides a snapshot of very recent LCS exposure and cannot be generalized to reflect adherence throughout the intervention or meaningfully quantify changes in total LCS intake during the study. It is also possible that the impacts of the COVID-19 pandemic on participants’ dietary intake and other diabetes management behaviors [38] may also limit the generalizability of the study findings. Finally, we also did not assess the participants’ Tanner stage at baseline and therefore were not able to account for the potential impacts of pubertal status on the study findings.

Despite these limitations, our study had several notable strengths, including the diverse sample of children with T1D and high rates of adherence to the intervention assessed using both self-report (daily LCS intake surveys) and objective (measurement of urinary sucralose and ace-K concentrations) methods. Our study was further strengthened by the collection of dietary and CGM data over one and two weeks, respectively, at both enrollment and follow-up. A particularly novel aspect of our study was the collection of photo-assisted food records, which increased the accuracy of the dietary data collected. In addition, this study is the first to examine the effects of LCS restriction on glycemic variability and cardiometabolic outcomes in children with T1D, among whom LCS are clinically recommended and widely consumed.

## 5. Conclusions

Children with T1D randomized to the intervention were adherent to 12 weeks of LCS restriction and parents of children in both groups indicated high study satisfaction and acceptability. Preliminary findings indicate that 12 weeks of LCS restriction did not impact glycemic variability or other cardiometabolic outcomes in children with T1D. The similar glycemic variability following 12 weeks of LCS restriction compared with usual LCS intake challenges whether reliance on LCS as a strategy to optimize blood glucose levels among children with T1D is justified, especially considering ongoing concerns about their consumption and limited research among youth. Further examination of the effects of LCS restriction among children with T1D in a larger trial is warranted to evaluate the generalizability and clinical relevance of these preliminary findings.

## Figures and Tables

**Table 1:** Baseline characteristics of participants randomized to LCS restriction or continuation of usual LCS intake (*n* = 28).

		All *n* = 28	LCS Restriction *n* = 15	Usual LCS Intake *n* = 13
Age, years (mean ± SD)		9.7 ± 2.0	9.7 ± 1.8	9.7 ± 2.3
Sex (*n*, %)				
	Female	14, 50%	7, 47%	7, 54%
	Male	14, 50%	8, 53%	6, 46%
Race (*n*, %)				
	White	14, 50%	8, 53%	6, 46%
	Black	12, 43%	6, 40%	6, 46%
	Mixed race	2, 7%	1, 7%	1, 8%
Ethnicity (*n*, %)				
	Hispanic	2, 7%	1, 7%	1, 8%
	Not Hispanic	26, 93%	14, 93%	12, 92%
Time since T1D diagnosis ^1^, years (mean ± SD)		4.2 ± 2.5	4.0 ± 2.9	4.4 ± 2.1
Usual LCS beverage intake ^2^ (*n*, %)				
	<1 cup per day ^3^	1, 4%	0, 0%	1, 8%
	1- < 2 cups per day	6, 21%	2, 13%	4, 31%
	≥2 cups per day	21, 75%	13, 87%	8, 62%
BMI percentile (mean ± SD)		67.6 ± 23.9	62.1 ± 29.0	73.9 ± 15.0
Energy intake ^4^, kcal (mean ± SD)		1860.6 ± 257.2	1864.7 ± 344.8	1856.0 ± 123.6
Total carbohydrate, g (mean ± SD)		233.6 ± 41.6	233.8 ± 56.7	233.5 ± 16.7
Total sugar, g (mean ± SD)		86.3 ± 24.2	87.0 ± 27.5	85.5 ± 21.8
Added sugar, g (mean ± SD)		55.6 ± 21.3	55.0 ± 21.9	56.2 ± 22.2
Total fat, g (mean ± SD)		77.5 ± 11.7	77.4 ± 15.8	77.6 ± 5.1
Total protein, g (mean ± SD)		65.2 ± 15.5	67.9 ± 16.3	62.2 ± 15.0
Dietary fiber, g (mean ± SD)		15.7 ± 3.0	16.4 ± 3.3	14.9 ± 2.6
Physical activity, days per week (mean ± SD)				
	Exercise or played so hard that your body got tired?	2.7 ± 2.0	2.4 ± 2.2	3.0 ± 1.7
	Exercised really hard for 10 min or more?	3.2 ± 2.2	2.6 ± 2.3	3.8 ± 1.9
	Exercised so much that you breathed hard?	2.4 ± 2.2	2.0 ± 2.5	2.8 ± 1.8
	So physically active that you sweated?	2.8 ± 2.3	2.6 ± 2.4	3.2 ± 2.2

^1^ Time since T1D diagnosis is based on *n* = 25 participants, as this information was not available for three participants. ^2^ Percentages do not sum to 100% due to rounding. ^3^ One participant reported usual consumption of less than 8 ounces of LCS sweetened beverages per day using the beverage questionnaire administered during the enrollment visit, despite reporting that they consumed ≥8 ounces of LCS sweetened beverages per day when screened for study eligibility. ^4^ Dietary data were available for seventeen participants, including nine in the LCS restriction (intervention) group and eight in the usual LCS intake (control) group. Note: No statistically significant differences in baseline characteristics were detected between the groups.

**Table 2:** Acceptability ratings based on parents’ responses to satisfaction survey, overall and by treatment group.

	All Participants (*n* = 25)	LCS Restriction (*n* = 13)	Usual LCS Intake (*n* = 12) ^1^
	N, % agree		
I would suggest this program to other parents of children with T1D	23, 92%	11, 85%	12, 100%
Overall, I feel that the program had a positive impact on my child’s diabetes management	23, 92%	11, 85%	12, 100%
I am more confident in my ability to manage my child’s diabetes after this program	18, 72%	9, 69%	9, 75%
Overall, I feel this program had reasonable expectations	21, 84%	12, 92%	9, 75%
Overall, I was glad I participated in this program	24, 96%	12, 92%	12, 100%
	N, % like		
Length of program	22, 88%	11, 85%	11, 92%
Length of each session with study staff	21, 84%	12, 92%	9, 75%
Frequency of text messages	21, 84%	12, 92%	9, 75%
Session scheduling process	24, 96%	13, 100%	11, 92%
Number of in-person sessions	23, 92%	13, 100%	10, 83%
Format and delivery of in-person sessions	25, 100%	13, 100%	12, 100%
Format and delivery of phone sessions	25, 100%	13, 100%	12, 100%
Format and delivery of online mid-point session	25, 100%	13, 100%	12, 100%

Note: % agree was calculated based on the number of participants who responded with a “4” agree or “5” strongly agree divided by the total number of participants. % like was calculated based on the number of participants who responded with a “4” like somewhat or “5” liked very much divided by the total number of participants. ^1^ One participant in the control group did not complete the satisfaction survey.

**Table 3:** Themes and subthemes identified in qualitative interviews with parents at the end of the study (*n* = 15).

Theme ^1^ Subtheme	Representative Quotations
**Facilitators of participation**	
Adherence to LCS restriction was not difficult ^2^	“I mean it wasn’t difficult—since we got put in the group where he couldn’t have any artificial sugars, I just didn’t buy them… he said he didn’t think it was hard at all”. “I think overall she just found it fairly manageable. I don’t know if she’d want to do it [avoid LCS] forever but she didn’t seem to have any issue with the time period that we did it over”.
Positive study experience	“We just had an overall good time. I mean it was fun being a part of it”. “I’m glad that we did it [the study]. You all are amazing… you try to take a difficult situation that we all find ourselves in, with having kids with type 1 diabetes and make it as positive as possible, and that is much appreciated”.
Financial compensation	“The best part was that it gave him something happy about having diabetes, because he got all the money”. “He was really excited that he got a little—he got the compensation”.
**Challenges of participation**	
Food records	“The food log was a little bit more challenging than I was envisioning at the beginning. I guess that I didn’t realize that putting down a sandwich was not enough and that I needed to put the bread the lettuce the tomato… itemize basically all the ingredients—that part I guess, I wasn’t prepared for that”. “I guess the one frustrating thing for me was the food logs, just because, keeping on top of that and trying to make sure we took pictures of everything and recording everything, that was my only complaint”.
In-person visits	“I was freaked out to go to the hospital [laughter]… so I was like okay, let’s go in and out as fast as we can… that was not a pleasant outing for me as a mom, you know, and we skipped the DXA because of that”. “The first visit here was nerve racking”.
Drinking only unsweetened beverages ^2^	“The only thing that was she didn’t like about the study was that she just was in the group where she had to drink the water cause she’s not a water drinker”. “She can disagree with me on this, but she does not like drinking water”.
Avoiding products with LCS ^2^	“Reading everything on the back [of the package] just to make sure… you guys gave us a list of things that are “code”, like no sugar… reading all that stuff just to make sure that you didn’t slip up was probably the hardest thing”. “It was hard because of the simple fact that you had to really read the labels and figure out what she can and cannot eat”.

^1^ Overarching themes are shown in bolded text. ^2^ Subthemes that were identified only among parents of children randomized to LCS restriction.

**Table 4:** Changes in glycemic variability and cardiometabolic biomarkers at 12 weeks compared with baseline, by treatment group.

	LCS Restriction (*n* = 13)		Usual LCS Intake (*n* = 13)	
	Baseline	Change	Baseline	Change
Time in range (%) ^1^	54.73 ± 5.96	2.00 ± 2.59	42.55 ± 6.14	3.91 ± 2.59
Time above range (%) ^1^	41.82 ± 6.46	−1.82 ± 2.88	54.00 ± 6.44	−4.09 ± 2.88
Time below range (%) ^1^	3.00 ± 0.76	0.15 ± 0.69	3.02 ± 0.89	0.03 ± 0.69
Average glucose (mmol/L) ^1^	9.44 ± 0.46	−0.16 ± 0.37	10.98 ± 0.94	−0.60 ± 0.37
SD glucose (mmol/L) ^1^	3.58 ± 0.16	−0.10 ± 0.14	3.97 ± 0.17	−0.03 ± 0.14
HbA1c (%) ^2^	7.72 ± 0.64	0.11 ± 0.23	9.26 ± 0.82	−0.29 ± 0.23
Total cholesterol (mmol/L) ^3^	4.60 ± 0.28	0.33 ± 0.19	4.56 ± 0.31	−0.31 ± 0.18 ^#,&^
LDL (mmol/L) ^3^	5.75 ± 0.47	0.45 ± 0.42	5.63 ± 0.51	−0.60 ± 0.39 ^&^
HDL (mmol/L) ^3^	3.55 ± 0.31	0.27 ± 0.15	3.40 ± 0.15	0.06 ± 0.14
FFA (mEq/L) ^4^	0.60 ± 0.10	0.33 ± 0.19	0.76 ± 0.10	−0.15 ± 0.18
Triglycerides (mmol/L) ^3^	2.88 ± 0.30	0.09 ± 0.35	3.89 ± 0.50	−0.90 ± 0.33 *
CRP (mg/L) ^5^	0.63 ± 0.17	−0.016 ± 0.98	2.33 ± 1.53	−1.60 ± 1.03
TNF-alpha (pg/mL) ^6^	1.03 ± 0.08	0.08 ± 0.07	1.41 ± 0.17	−0.23 ± 0.08 *^,#,&^

SD = standard deviation; HbA1c = hemoglobin A1c; LDL = low-density lipoprotein; HDL = high-density lipoprotein; FFA = free fatty acids; CRP = C-reactive protein; TNF-alpha = tumor necrosis factor alpha. All values are presented as mean ± standard error. There were no statistically significant differences in baseline values between the groups. * *p* < 0.05, indicates a statistically significant within-group difference at 12 weeks compared to baseline. ^#^ *p* < 0.05, indicates a statistically significant difference in the pre/post change in the intervention group compared with the control group prior to adjustment for relevant covariates. ^&^ *p* < 0.05, indicates a statistically significant difference in the intervention group compared with the control group after adjustment for age, sex, race, and change in BMI (except for models where BMI is the outcome). ^1^ Based on *n* = 22 participants (*n* = 11 in the intervention group and *n* = 11 in the control group), as one participant did not have available CGM data and three participants did not have reliable CGM data. ^2^ Based on *n* = 23 participants (*n* = 12 in the intervention group and *n* = 11 in the control group) who provided a blood sample at baseline. ^3^ Based on *n* = 15 participants (*n* = 7 in the intervention group and *n* = 8 in the control group) who were fasted at the time of blood sample collection. ^4^ Based on *n* = 15 participants (*n* = 7 in intervention group, *n* = 8 in control group) with FFA data available at both timepoints. ^5^ Based on *n* = 23 participants (*n* = 12 in intervention group, *n* = 11 in control group) with CRP data available at both timepoints. ^6^ Based on *n* = 22 participants (*n* = 12 in intervention group, *n* = 10 in control group) with TNF-alpha data available at both timepoints.

**Table 5:** Change in dietary intake at 12 weeks compared with baseline, by treatment group.

	LCSB Restriction (*n* = 9)		Usual LCS Intake (*n* = 8)	
	Baseline	Change	Baseline	Change
Energy intake (kcal)	1864.7 ± 114.93	−190.8 ± 106.40	1855.9 ± 43.70	−245.3 ± 112.90 *
Carbohydrate (% kcal)	49.7 ± 1.62	1.7 ± 2.46	50.6 ± 1.11	−8.5 ± 2.61 *^,#,&^
Total sugar (% kcal)	18.8 ± 1.62	−0.7 ± 1.69	18.7 ± 1.55	−2.5 ± 1.79
Added sugar (% kcal)	11.6 ± 1.15	−0.5 ± 1.52	12.4 ± 1.65	−1.2 ± 1.61
Fat (% kcal)	37.4 ± 1.88	−0.9 ± 1.91	37.3 ± 0.50	5.2 ± 2.02 *^,#^
Protein (% kcal)	14.9 ± 0.97	−1.4 ± 1.10	13.5 ± 0.83	3.2 ± 1.16 *^,#,&^
Dietary fiber (g)	16.4 ± 1.10	−1.9 ± 1.53	14.9 ± 0.91	−2.1 ± 1.62

All values are presented as mean ± standard error. There were no statistically significant differences in baseline values between the groups. Note: Based on *n* = 17 participants (*n* = 9 in intervention group, *n* = 8 in control group) with dietary data available at both timepoints. * *p* < 0.05, indicates a statistically significant within-group difference at 12 weeks compared to baseline. ^#^ *p* < 0.05, indicates a statistically significant difference in the pre/post change in the intervention group compared with the control group prior to adjustment for relevant covariates. ^&^ *p* < 0.05, indicates a statistically significant difference in the intervention group compared with the control group after adjustment for age, sex, race, and change in BMI (except for models where BMI is the outcome).

## Data Availability

The data will be made available upon reasonable request to the corresponding author.

## Supplementary Materials

The following supporting information can be downloaded at: https://www.mdpi.com/article/10.3390/nu15183867/s1, Supplementary Table S1: Urinary sucralose and acesulfame-potassium concentrations measured by LCS-MS at baseline and week 12, by treatment group. Supplementary Table S2: Changes in glycemic variability and cardiometabolic biomarkers at 12 weeks compared with baseline, by treatment group, in sensitivity analyses excluding non-adherent participants. Supplementary Table S3: Change in body composition at 12 weeks compared with baseline, by treatment group. Supplementary Table S4: Change in dietary intake at 12 weeks compared with baseline, by treatment group, in sensitivity analyses excluding non-adherent participants.
