# Supplementary material for: Effects of Low-Calorie Sweetener Restriction on Glycemic Variability and Cardiometabolic Health in Children with Type 1 Diabetes: Findings of a Pilot and Feasibility Study
Source: Nutrients. 2023 Sep 5;15(18):3867. doi: 10.3390/nu15183867 (PMC10534616; doi:10.3390/nu15183867)
Supplement: Supplementary file 1 [file nutrients-15-03867-s001.zip › nutrients-2566753-supplementary.pdf]

Supplemental Table S1. Urinary sucralose and acesulfame-potassium concentrations measured by LCS-MS at baseline and week 12, by treatment group

	LCS Restriction (n=12)		Usual LCS Intake (n=13)	
	Baseline	Week 12	Baseline	Week 12
**All participants[1]**				
Sucralose (ng/mL)	1,219 ± 653	13 ± 6.9	615 ± 269	1,146 ± 619
Acesulfame-potassium (ng/mL)	37,740 ± 13,825	9,080 ± 8,392	26,700 ± 11,829	63,079 ± 34,978
	**LCS Restriction (n=11)**		**Usual LCS Intake (n=12)**	
	Baseline	Week 12	Baseline	Week 12
**Excluding non-adherent participants[2]**				
Sucralose (ng/mL)	1,330 ± 705	14 ± 7.4	654 ± 289	1,242 ± 664
Acesulfame-potassium (ng/mL)	40,118 ± 14,919	698 ± 453	28,925 ± 12,630	68,335 ± 37,593

All values are presented as mean ± standard error.

[1]One participant in the LCS restriction group was unable to provide a urine sample at both timepoints and was therefore excluded fro the analysis.

[2]Two participants (one randomized to LCS restriction and one randomized to usual LCS intake) indicated lack of adherence to treatment assignment based on their daily LCS questionnaire responses.

Supplemental Table S2. Changes in glycemic variability and cardiometabolic biomarkers at 12 weeks compared with baseline, by treatment group, in sensitivity analyses excluding non-adherent participants

	LCSB Restriction (n=12)		Usual LCS Intake (n=12)	
	Baseline	Change	Baseline	Change
Time in range (%)[1]	57.00 ± 6.09	0.80 ± 2.57	40.30 ± 6.32	5.00 ± 2.57
Time above range (%)[1]	39.30 ± 6.58	-0.50 ± 2.70	56.90 ± 6.36	-5.80 ± 2.70
Time below range (%)[1]	3.19 ± 0.82	0.10 ± 0.59	2.32 ± 0.55	0.63 ± 0.59
Average glucose (mmol/L)[1]	9.35 ± 0.5	-0.1 ± 0.39	11.27 ± 0.99	-0.76 ± 0.39
SD glucose (mmol/L)[1]	3.57 ± 0.18	-0.12 ± 0.15	3.96 ± 0.18	-0.06 ± 0.16
HbA1c (%)[2]	7.72 ± 0.70	0.11 ± 0.24	9.52 ± 0.86	-0.35 ± 0.25
Total cholesterol (mmol/L)[3]	4.58 ± 0.28	0.33 ± 0.19	4.56 ± 0.31	-0.31 ± 0.18[#,&]
LDL (mmol/L)[3]	5.75 ± 0.47	0.45 ± 0.42	5.63 ± 0.51	-0.6 ± 0.39
HDL (mmol/L)[3]	3.55 ± 0.31	0.27 ± 0.15	3.40 ± 0.15	0.06 ± 0.14
FFA (mEq/L)[4]	0.60 ± 0.10	0.33 ± 0.19	0.76 ± 0.10	-0.15 ± 0.18
Triglycerides (mmol/L)[3]	2.88 ± 0.3	0.09 ± 0.35	3.89 ± 0.5	-0.90 ± 0.33[*]
hsCRP (mg/L)[5]	0.63 ± 0.17	-0.02 ± 1.01	2.41 ± 1.69	-1.63 ± 1.10
TNF-alpha (pg/mL)[6]	1.03 ± 0.08	0.08 ± 0.06	1.32 ± 0.16	-0.16 ± 0.07[*,#,&]

All values are presented as mean ± standard error

[*]p<0.05, indicates statistically significant within-group difference at 12-weeks compared to baseline.

[#]p<0.05, indicates statistically significant difference in the pre/post change in the intervention group compared with the control group prior to adjustment for relevant covariates.

[&]p<0.05, indicates statistically significant difference in the intervention group compared with the control group after adjustment for age, sex, race, and change in BMI (except for models where BMI is the outcome).

[1]Based on n=20 participants (n=10 in the intervention group and n=10 in the control group), as one participant did not have available CGM data and three participants did not have reliable CGM data.

[2]Based on n=21 participants (n=11 in the intervention group and n=10 in the control group) who provided a blood sample at baseline.

[3]Based on n=15 participants (n=7 in the intervention group and n=8 in the control group) who were fasted at the time of blood sample collection.

[4]Based on n=15 participants (n=7 in intervention group, n=8 in control group) with FFA data available at both timepoints.

[5]Based on n=22 participants (n=12 in intervention group, n=10 in control group) with CRP data available at both timepoints.

[6]Based on n=21 participants (n=12 in intervention group, n=9 in control group) with TNF-alpha data available at both timepoints.

Supplemental Table S3. Change in body composition at 12 weeks compared with baseline, by treatment group

	LCSB Restriction (n=3)		Usual LCS Intake (n=5)	
	Baseline	Change	Baseline	Change
Body fat (%)	30.3 ± 3.2	0.1 ± 1.1	32.6 ± 1.9	1.0 ± 0.8
Fat mass (g)	10158.1 ± 2514.9	-163.5 ± 547.2	12952.7 ± 3417.4	987.8 ± 423.8

Based on n=8 participants who had a DXA scan at both timepoints.
All values are presented as mean ± standard error.
No statistically significant differences were detected within or between groups.
Models for body composition outcomes are unadjusted models due to small sample size.

Supplemental Table S4. Change in dietary intake at 12 weeks compared with baseline, by treatment group, in sensitivity analyses excluding non-adherent participants

	LCSB Restriction (n=8)		Usual LCS Intake (n=8)	
	Baseline	Change	Baseline	Change
Energy intake (kcal)	1926.5 ± 109.88	-200.5 ± 116.6	1855.9 ± 43.70	-245.3 ± 116.6
Carbohydrate (% kcal)	50.5 ± 1.57	1.7 ± 2.70	50.6 ± 1.11	-8.5 ± 2.70[*,#,&]
Sugar (% kcal)	19.6 ± 1.59	-0.4 ± 1.84	18.7 ± 1.55	-2.5 ± 1.84
Added sugar (% kcal)	12.2 ± 1.16	-0.6 ± 1.66	12.4 ± 1.65	-1.2 ± 1.66
Fat (% kcal)	36.6 ± 1.88	-0.8 ± 2.09	37.3 ± 0.50	5.2 ± 2.09[*]
Protein (% kcal)	15.0 ± 1.09	-1.4 ± 1.20	13.5 ± 0.83	3.2 ± 1.20[*,#,&]
Dietary fiber (g)	17.0 ± 1.08	-1.8 ± 1.67	14.9 ± 0.91	-2.1 ± 1.67

Note: Based on n=16 participants (n=8 in intervention group, n=8 in control group) who were adherent to the intervention and had dietary data available at both timepoints.
All values are presented as mean ± standard error.
[*]p<0.05, indicates statistically significant within-group difference at 12-weeks compared to baseline.
[#]p<0.05, indicates statistically significant difference in the pre/post change in the intervention group compared with the control group prior to adjustment for relevant covariates.
[&]p<0.05, indicates statistically significant difference in the intervention group compared with the control group after adjustment for age, sex, race, and change in BMI (except for models where BMI is the outcome).
